# A multi-Omic resource for exploring microbial eukaryotes in the meromictic freshwater Lake Pavin

**DOI:** 10.1038/s41597-026-06573-0

**Published:** 2026-01-14

**Authors:** Damien Courtine, Cécile Lepère, Ivan Wawrzyniak, Anne Moné, Hermine Billard, Jonathan Colombet, Arthur Monjot, Corinne Cruaud, Corinne Da Silva, Jean-Marc Aury, Didier Debroas, Gisèle Bronner

**Affiliations:** 1 Laboratoire Microorganismes: Génome et Environnement, Université Clermont Auvergne, CNRS, Clermont-Ferrand, France; 2 UMR6538 GEO OCEAN, Université de Bretagne Occidentale, CNRS, IRD, Ifremer, 29280 Plouzané France; 3 Institut de Biologie François Jacob, Genoscope, CEA, Université Paris-Saclay, Evry, France; 4 Génomique Métabolique, Genoscope, Institut François Jacob, CEA, CNRS, Univ Evry, Université Paris-Saclay, Evry, France

**Keywords:** Water microbiology, Microbial ecology, Data processing

## Abstract

Although recent advances in high-throughput sequencing have greatly expanded our understanding of microbial diversity and function in aquatic ecosystems, progress in studying freshwater microbial eukaryotes has been more limited, mainly due to their large genomes, immense diversity, and largely uncharacterised physiologies. In this work, we present a comprehensive multi-omic dataset, eukaryote-centred, including targeted-metagenomic (18S rDNA V4 and V9), metagenomic, metatranscriptomic and single amplified genomes (SAGs). Both the oxic and anoxic layers of Lake Pavin (France), a permanently stratified freshwater lake, were sampled at four distinct times throughout 2018, by day and night, targeting microbial eukaryotes of two size classes (0.65–10 µm and 10–50 µm). This dataset comprises 106 eukaryotic metagenome-assembled genomes (MAGs), over 9 million unigenes and 11 SAGs, encompassing several under-represented taxa in public databases (*e.g*. Perkinsea, Chytridiomycota, Cryptista). Altogether, this dataset represents a resource for exploring the functional diversity and spatio-temporal dynamics of microbial eukaryotes.

## Background & Summary

As reservoirs of microbial biodiversity, aquatic ecosystems play a central role in global biogeochemical cycles. However, the specific and functional diversity of microorganisms inhabiting these ecosystems remains largely unknown^1^ because a significant proportion of these microbes are uncultivable using traditional laboratory methods. This is particularly true for microbial eukaryotes, which have complex metabolisms and whose physiologies may vary upon environmental conditions^2^. Recent advances in high-throughput sequencing technologies and multi-omics approaches have revolutionised the study of these communities. The complementarity of these approaches, whether targeted (amplicon-based) or untargeted (metagenomics, metatranscriptomics), offers a more comprehensive view of microbial communities within an ecosystem. This includes their taxonomic composition, genetic potential, gene expression patterns, and functional activities, thereby revealing the complexity of interactions among these microorganisms.

However, non-targeted approaches are rarely used for the study of microbial eukaryotes, largely due to the complexity involved in analysing their diverse and often large genomes, as well as the challenges in interpreting their functional roles within ecosystems. A few pioneering eukaryote-focused metagenomic studies have been conducted in ocean ecosystems^3,4^, however similar comprehensive investigations in freshwater environments remain scarce^5,6^.

**Fig. 1:**
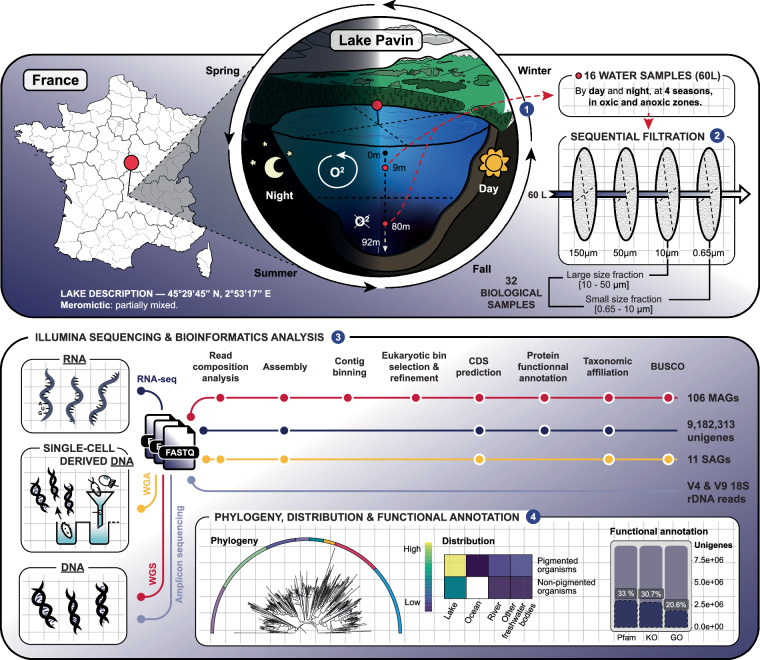
Graphical overview of the sampling, data production and analysis. Top, Lake Pavin sampling conditions (2018) and size class filtering strategy. Bottom, bioinformatics analysis for each approach: amplicon based-, metagenomic, metatranscriptomic, and single-cell amplified genome sequencing. Synthetic overview of the data is provided by placing the MAGs and SAGs in an evolutionary framework, and representing their distribution in different aquatic environments via a heatmap. The functional potential of the microbial communities can be derived from the annotation of the unigenes catalogue as found in Monjot *et al*.^10^.

Focusing on the meromictic Lake Pavin^7^ and its microbial eukaryotic community, we provide an exhaustive -omic dataset that includes amplicon-based (18S rDNA regions V4 and V9), metagenomic and metatranscriptomic datasets, as well as metagenome-assembled genomes (MAGs) and single amplified genomes (SAGs). The Lake Pavin is located in Auvergne (France), characterised by a mixed oxygenated upper layer (top to *ca*. 60 m) and a permanent anoxic deep layer (*ca*. 60–92 m; Fig. 1). Water sampling was carried out in both layers (at 9 m and 80 m) by day and night, in April, June, September, and November, and for two size classes (0.65–10 and 10–50 µm).

**Fig. 2:**
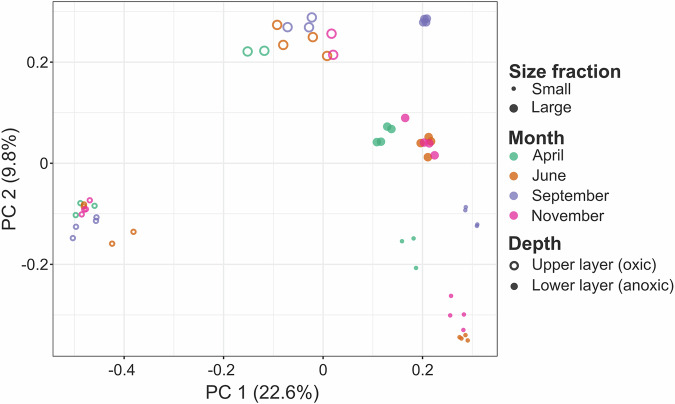
Principal Coordinates Analysis of the metagenomes. Relations between the metagenomic samples based on a Bray-Curtis distance matrix of their sequence composition as measured with SimkaMin^30^ on 50 million reads and 10 million k-mers per sample.

**Fig. 3:**
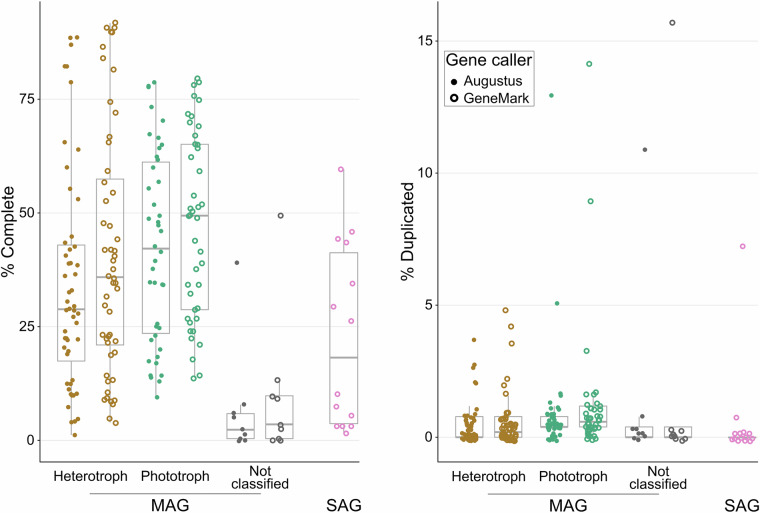
BUSCO completion and redundancy scores of the MAGs and SAGs based on the eukaryota_odb10 single-copy core genes set. Completion is the proportion of *unique + duplicated* genes found; redundancy is the proportion of *duplicated* genes.

**Fig. 4:**
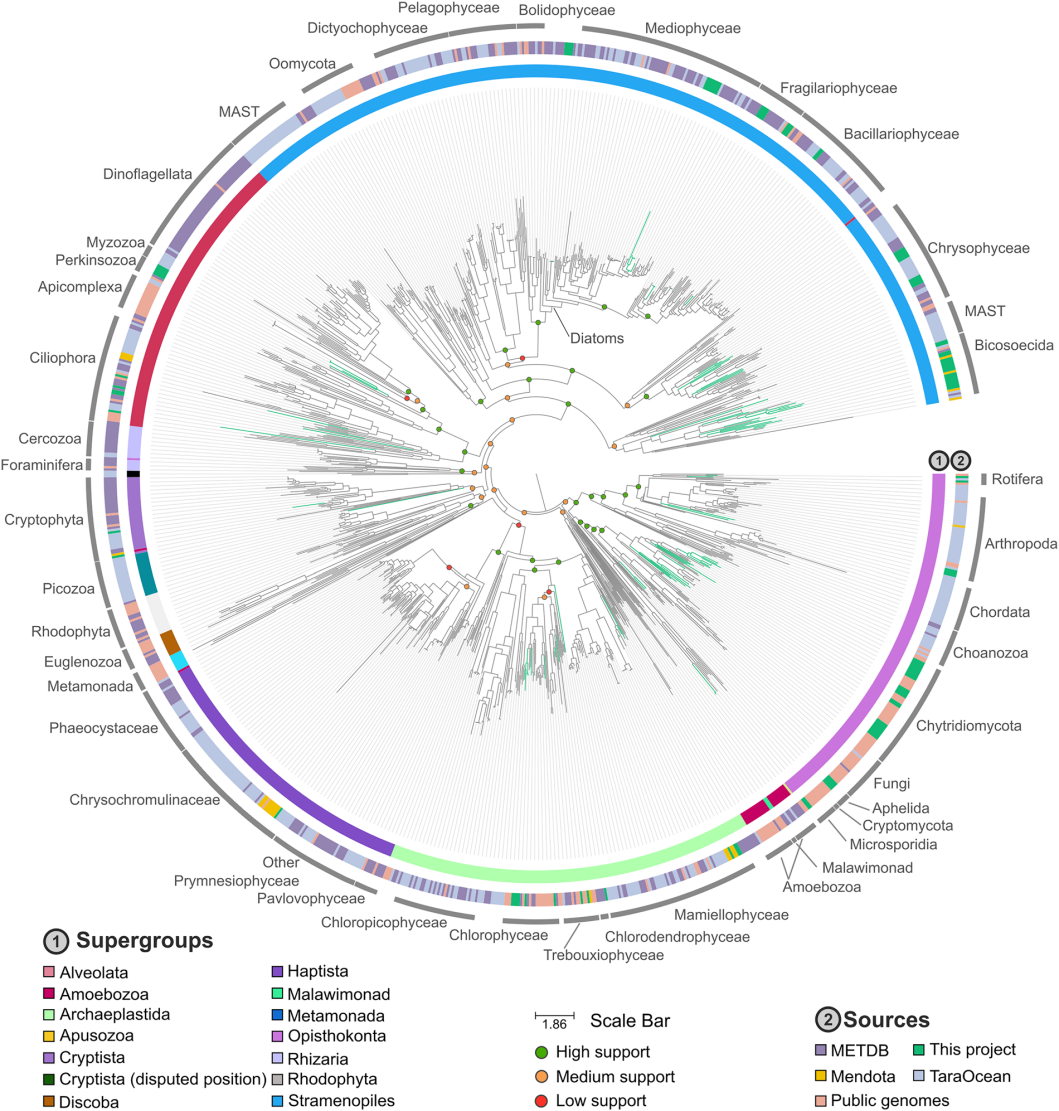
Phylogenetic tree of the concatenated DNA-dependent RNA polymerase protein sequences from eukaryotes. The maximum likelihood phylogenetic tree of the concatenated sub-units from the DNA-dependent RNA polymerase, 6 genes in total, including MAGs and SAGs of this work, Tara Ocean MAGs^3^, METdb transcriptomes^40^, genomes from the public databases and MAGs from the Lake Mendota^5^, was generated using 7,659 sites in the alignment and the LG + F + I + R10 model. The clade of Opisthokonta was used as the outgroup. Phylogenetic supports were considered high (aLRT ≥ 80 and UFBoot ≥ 95), medium (aLRT ≥ 80 or UFBoot ≥ 95), or low (aLRT < 80 and UFBoot < 95).

**Fig. 5:**
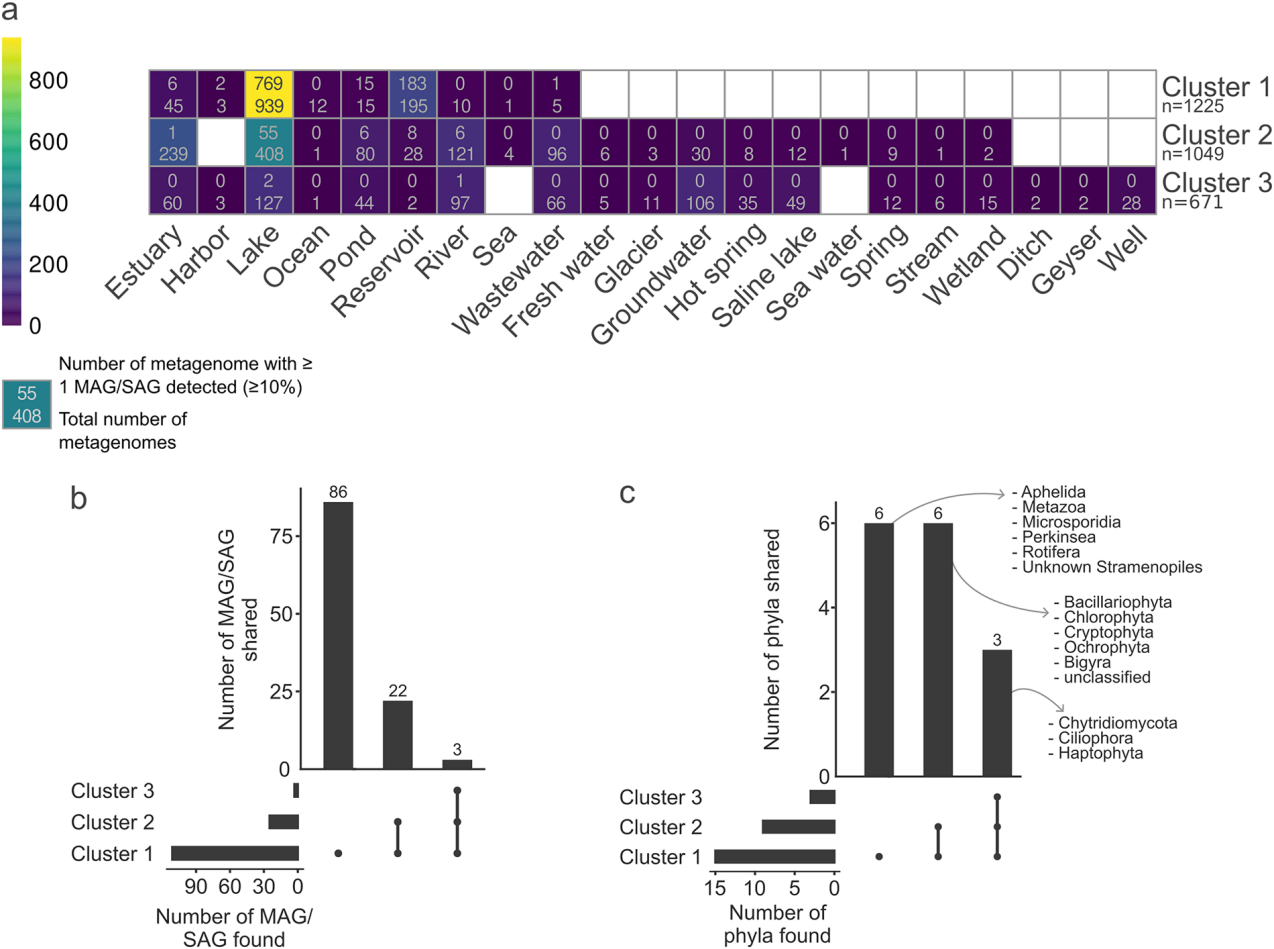
Distribution of MAGs and SAGs in water metagenomes. a, Detection of MAGs and SAGs per metagenomic dataset. These datasets were clustered based on the number of read (CLR transformation) mapping on the MAGs and SAGs. Each square summarises the number of metagenomes that belong to an ecosystem type (bottom) and the number of metagenomes in which at least one MAG or SAG was detected (top). A genome is considered detected in a metagenome when its breadth of coverage is ≥10%. Empty square means no data. b, UpSet plot that describes the clusters composition in term of number of MAG or SAG detected, *i.e*. at least one genome with a breadth of coverage ≥10% in ≥1 metagenome. c, UpSet plot of the phyla present in the different clusters. for b and c, empty sets are not shown.

Targeted metagenomic (amplicon-based) data are provided as raw reads. Whole genome shotgun metagenomic data, with a total of 8,8 billion read pairs distributed in 57 samples (277 million read pairs on average; Table 1), were partitioned and assembled according to size class and oxygen status (Fig. 2), leading to 2.8 million contigs of size greater than 2.5 kb, representing 19.9 Gbp. The binning of these contigs produced 5,657 bins, 106 of which were identified as putative eukaryotes, and manually refined using Anvi’o^8^ to obtain MAGs. Gene predictions were used to estimate MAG completeness which is 39.9% on average (Fig. 3). Among these MAGs, 52 were classified as heterotrophs, including 25 identified as confirmed or potential parasites (Chytridiomycota, Aphelida, Perkinsea and Microsporidia). Additionally 42 MAGs belonged to pigmented eukaryotes, mostly diatoms (Bacillariophyta) and Chlorophyta (Fig. 4). About 10% of the MAGs were assembled from the deep anoxic layer. These MAGs were complemented by 11 SAGs with an average completion of 22.7% (maximum 59.6%, Fig. 3) affiliated to Perkinsea, Chytridiomycota, Aphelida and Bacillariophyta. To assess how representative our dataset is, we mapped metagenomic reads from diverse water bodies worldwide onto the Lake Pavin MAGs and SAGs. Clustering based on Aitchison distance calculated from transformed read counts grouped the metagenomes onto three distinct clusters. Lake Pavin MAGs and SAGs are mostly detected in metagenomes associated with lentic ecosystems like lakes or reservoirs (Fig. 5a,b) and 25 MAGs, including 22 from pigmented taxa, were found across a variety of ecosystems, such as rivers, ponds or estuaries with some marginal presence in coastal environments (harbour, estuary; Fig. 5a,c). These findings underline the specificity of the MAGs and SAGs presented in this study for freshwater environments, although pigmented taxa appear to inhabit a broader range of ecosystems compared to non-pigmented taxa.

**Table 1:** Summary of data generated per type, in million pairs of reads.

Month	Zone	Size fraction	Period of the day	Metagenomic	Metatranscript-omic	Metabarcoding 18S V4	Metabarcoding 18S V9
April	Oxic	Small	Day	305	289	0.549	0.678
			Night	127*	262	0.227*	0.219*
		Large	Day	332	341	0.281*	0.386
			Night	339	332	0.494	0.440
	Anoxic	Small	Day	341	174*	0.525	0.238
			Night	126*	180*	0.215*	0.128*
		Large	Day	232*	179*	0.486	0.263*
			Night	193*	186*	0.215*	0.243*
June	Oxic	Small	Day	287	239	0.455	0.273
			Night	371	297	0.486	0.312
		Large	Day	378	233*	0.408	0.419
			Night	341	226*	0.478	0.267
	Anoxic	Small	Day	350	304	0.441	0.333
			Night	385	193*	0.397	0.443
		Large	Day	341	219*	0.459	0.249
			Night	263	199*	0.419	0.478
September	Oxic	Small	Day	453	325	0.406	0.306
			Night	241	326	0.397	0.246
		Large	Day	304	156*	0.496	0.248
			Night	347	132*	0.452	0.234
	Anoxic	Small	Day	235	242	0.440	0.342
			Night	268	98*	0.405	0.543
		Large	Day	164*	144*	0.297*	
			Night	262	120*	0.460	0.220
November	Oxic	Small	Day	289	389	0.373	0.250
			Night	307	371	0.452	0.652
		Large	Day	344	367	0.479	0.433
			Night	189	370	0.473	0.358
	Anoxic	Small	Day	280	317	0.435	0.404
			Night	251	345	0.540	0.398
		Large	Day	106*	184*	0.278*	0.142*
			Night	100*	178*	0.248*	0.196*
Total read pairs				**8,853**	**7,918**	**13.16**	**10.34**
Average read pairs per biological sample				**277**	**247**	**0.41**	**0.33**
Number of samples sequenced				**57**	**48**	**57**	**56**

The numbers reflect the deposited data, after quality control from the sequencing platform. The asterisk (*) indicates samples with merged duplicates.

The metatranscriptomic data, spanning 48 samples with on average 247 million read pairs (Table 1), were processed into unigenes and annotated as previously described in Carradec *et al*.^9^ and Monjot *et al*.^10^ respectively. Similarity analysis of these data revealed differences in expression profiles between size classes, oxic-anoxic layer and sampling periods (ANOSIM, *p* < 0.001), whereas not between day and night samples^10^. Coupled with a trait-based approach, amplicon-based and metatranscriptomic data were used to investigate the metabolic potential and genetic signatures of ecologically meaningful functional groups of microbial eukaryotes (*e.g*. phototrophs, heterotrophs, saprotrophs, parasites, mixoplankton)^10,11^. These analyses revealed a remarkable diversity in the anoxic zone of Lake Pavin as well as strong seasonal variations in the oxic layer, particularly among parasitic and mixoplanktonic microorganisms. Parasites benefited from water mixing events in spring and autumn, while mixoplankton, whose metabolic versatility was confirmed by the global analysis of functional genes, took advantage of their phagotrophic abilities during nutrient limitation specific to winter.

Overall, this dataset offers a more comprehensive genetic characterisation of the microbial eukaryotic community within a lake ecosystem featuring contrasting environmental conditions. These data complement marine datasets^3,4^ and offers new resources that are still rare in databases, such as MAGs from freshwater environments and anoxic zones. Moreover, the inclusion of genomes from under-represented taxa in this environment enriches the available resources for studying protist diversity^12^. Additionally, this integrated dataset can serve as a valuable benchmark for the development and validation of future analytical tools and methodologies.

## Methods

### Sample collection

Water samples were collected at the centre of the Lake Pavin (Massif Central, France, 45°29′45″ N, 2°53′17″ E) in April, June, September, and November of the year 2018. For each sampling event, a total of 60 L of water were collected using a Von Dorn bottle from the oxygenated layer (9 m) and the permanently anoxic lower layer (80 m), once during the day and once at night. The samples were pre-filtered sequentially on nylon filter mesh (sifting fabric) with decreasing pore-size (150 µm, 50 µm and 10 µm). Microorganisms retained on the 10 μm filter, representing the large size class (10–50 μm), were resuspended in a final volume of 300 mL of sterile water. Those corresponding to the small size class (<10 μm) were concentrated using tangential flow filtration with a Spectrum KrosFlo PES 0.65 μm column (Spectrum Labs, Rancho Dominguez, CA, USA) and collected in a final volume of 1 L. Given that filtration-based sampling can selectively impact microbial cell recovery due to variations in size, morphology, or adhesion properties, we carefully minimised handling time and optimised resuspension protocols to mitigate such biases. Samples were treated with 0.01% pluronic acid and centrifuged at 10,000 g, 4 °C for 30 min. The pellets were then divided for DNA and RNA extraction with the aim to sequence all samples in duplicates. The pellets were resuspended in 250 μL of RLT buffer for DNA extraction (Qiagen, Hilden, Germany) and with the addition of β-mercaptoethanol for RNA extraction. Samples were immediately frozen in liquid nitrogen.

DNA samples were disrupted by bead beating for 30 s at 30 Hz, three times, with 0.1 g of 0.1 mm glass beads (Sigma-Aldrich, Saint-Louis, MO, USA), followed by 1 min of centrifugation at 800 g. The supernatants were adjusted to 300 μL with extraction buffer (50 mM glucose, 10 mM Ethylenediaminetetraacetic acid (EDTA), 25 mM Tris) and incubated with 150 μL of 10% sodium dodecyl sulfate (SDS) for 5 min. Then, 5 μL of proteinase K (10 mg/mL) and 5 μL of RNAse A (10 U/μL) were added. The mixture was incubated for 2 h at 37 °C. Then, 80 μL of cetyltrimethylammonium bromide (CTAB) solution (10%, NaCl 0.7 M) and 100 μL of NaCl 5 M were added before the nucleic acid extraction by phenol-chloroform-isoamyl alcohol (same volume as sample, ratio 25:24:1 v/v/v) and isopropanol precipitation. DNA pellets were washed with 70% ethanol and resuspended in 30 μL of water and stored in LoBinding DNA tubes.

The total RNA extraction were processed with the RNeasy Mini kit (Qiagen) with some modifications. Samples were disrupted by bead beating for 30 s at 30 Hz, three times, and the supernatant was kept. The sample was subjected to a second bead beating round, same conditions, with 400 μl of RLT buffer with 1% 2-mercaptoethanol and 0.1 g of 0.1 mm glass beads (Sigma-Aldrich). Both supernatants were pooled. The end of the protocol was carried out according to the manufacturer’s recommendations. Total RNA was eluted in 30 μL of RNAse free water^10^.

Several samples, particularly those from the anoxic zone, did not yield sufficient DNA/RNA for sequencing. We therefore had to pool the duplicates, and ultimately obtained 57 DNA and 48 RNA samples (Table 1).

### Libraries preparation and sequencing

The V4 region of the 18S rDNA gene was PCR amplified using the 515 F (5′-GTGYCAGCMGCCGCGGTA-3′)^13^ and 951 R (5′-TTGGYRAATGCTTTCGC-3′)^14^ primers and subsequently purified as presented in Chauvet *et al*.^15^. The V9 region was amplified using the universal eukaryotic forward primer 1389 F (5′-TTGTACACACCGCCC-3′)^16^ and reverse primer 1510 R (5′-CCTTCYGCAGGTTCACCTAC-3′)^17^. Starting from 2.5 to 10 ng of DNA, three PCR reactions were performed for each sample (98 °C for 30 s followed by 25 cycles 10 s-98 °C, 30 s-53 °C, 30 s-72 °C and a final extension 10 min-72 °C) with the Phusion High Fidelity PCR Master Mix with GC buffer (Thermo Fisher Scientific, Waltham, MA, USA). Then, both 18S rDNA amplicons (V4 and V9) were processed with the same methods: PCR products were pooled by sample, purified using 1X AMPure XP beads (Beckman Coulter, Brea, CA, USA) and quantified with a Fluoroskan Microplate Fluorometer (Thermo Fisher Scientific). A size profile analysis was performed with a LabChip GX Nucleic Acid Analyzer (Revvity, Waltham, MA, USA). A library was prepared for each amplicon, using the NEBNext DNA Modules Products (New England Biolabs, Ipswich, MA, USA) and NEXTFLEX Unique Dual Index Barcodes (Perkin Elmer, Waltham, MA, USA). Ligated products were amplified using the Kapa Hifi HotStart NGS library Amplification kit (Roche, Basel, Switzerland), followed by 1X AMPure XP (Beckman Coulter) clean up.

Metatranscriptomic libraries were prepared from 100 ng of total RNA with the NEBNext Ultra II Directional RNA Library Prep Kit (New England Biolabs)^11^. The metagenomic libraries were prepared using the NEBNext Ultra II kit (New England Biolabs) using 2.4 to 250 ng of DNA.

All libraries were sequenced at the Genoscope (Évry, France) with different Illumina systems (Illumina, San Diego, CA, USA): the V4 and V9 amplicons were sequenced on a HiSeq 2500 (2 × 250 bp) and MiSeq (2 × 150 bp) respectively, while both metagenomic and metatranscriptomic libraries were sequenced on a NovaSeq 6000 (2 × 150 bp).

### Meta-omic reads assembly and binning

Metatranscriptomic reads were assembled to form unigenes as described in Carradec *et al*.^9^, and were further functionally and taxonomically annotated as described in Monjot *et al*.^11^. Paired-end reads from 48 samples (Table 1) were assembled on a per sample basis using Velvet^18^ v1.2.07 (k-mer size of 89). Contigs smaller than 150 bp were removed, and isoforms were detected using Oases^19^ v0.2.08. Contig redundancy was reduced by clustering with CD-HIT-EST^20^ v4.6.1 (-id 95 -aS 90), retaining the longest contig as the unigene reference sequence. This unigene catalogue was analysed with TransDecoder^21^ v5.5.0 to identify putative proteins of a minimum length of 70 amino acids. Predicted proteins were screened against the AntiFam^22^ database v7.0 using hmmsearch^23^ with the --cut_ga parameter and functionally annotated according to KEGG using KoFamScan^24^ v1.3.0 (HMM profiles release 2022-01-03, e-value < 1e-5) and Pfam-A^25^ v35.0 using hmmsearch and the --cut_ga parameter. The GO terms were extracted from the Pfam annotation using the Gene Ontology cross-reference classification system^26^. Taxonomic affiliations were inferred by comparing predicted proteins to the database associated to MetaEuk^27^ using MMseqs2^28^ (v407b315, mmseqs taxonomy --tax-lineage 1 --lca-mode 2 --max-seqs 100 -e 0.00001 -s 6 --max-accept 100). Proteins and associated unigenes affiliated to Human, Bacteria, Viruses and Metazoans were excluded from the catalogue. Starting from 7.9 billion read-pairs, this pipeline produced in 9,926,643 unigenes and 9,182,313 proteins, of which 30.7% were assigned a KO annotation, and 30.8% were affiliated to the eukaryotic domain. Overall, 1,280,290 unigenes have both taxonomic and functional annotation. Expression profiles of unigenes were determined by mapping reads from each sample to the unigenes reference sequences using bwa^29^ v0.7.15 (bwa aln -l 30 -O 11 -R 1; bwa sampe -a 20000 -n1 -N) followed by duplicate removal (samtools rmdup). Read counts were then restricted to alignments with at least 80% of the read length aligned and a minimum of 95% sequence identity.

For metagenome reads, the 57 samples (8.8 billion read pairs; Table 1) were first subjected to reads composition analysis using SimkaMin^30^ on 50 M reads and 10 M k-mers per sample. The PCoA on a Bray-Curtis distance of the samples reads composition highlighted sample clustering by size class and oxygen status (Fig. 2). Grouping samples according to these parameters, 4 co-assemblies were realised with MEGAHIT^31^ v1.2.9 and parameters --k-list 3, 47, 59, 79, 99, 119, 141 --min-contig-len 1000. Contigs smaller than 2.5 kb were removed and the assemblies were then processed using the Anvi’o^8^ framework v8 for gene prediction with Prodigal^32^ v2.6.3. The short-reads were then mapped on the contigs with bwa mem^33^ v0.7.17, results were processed with Samtools^34^ to generate BAM files. Alignments were filtered by msamtools^35^ v1.1.0 to keep reads with more than 95% identity over a minimum of 75 nt. Anvi’o facilities were used to combine all BAM files within each co-assembly to estimate per sample contigs abundances and run MetaBAT2^36^ with default parameters for binning. Candidate eukaryotic bins were identified by the presence of 83 single-copy core genes^37^ (anvi-estimate-genome-completeness) and were subjected to manual inspection via the Anvi’o interactive interface (command anvi-refine) to remove spurious contigs, based on either “sequence composition” divergence or “differential coverage”. Bins completeness and redundancy were estimated with Anvi’o (anvi-summarize). This process resulted in 106 eukaryotic candidate MAGs of which 103 were retained for advanced gene prediction. As suggested by Schoenle *et al*.^12^, the 5,551 unrefined bins may correspond to organisms that were in association with microbial eukaryotes (*e.g*. symbiosis, parasitism, predation). It could also be eukaryote genomes with lower completion, that were not detected because of an insufficient coverage of the eukaryote marker gene set during the analysis.

### MAGs gene prediction

As eukaryotic gene prediction remains a challenging task, all current gene prediction tools use intrinsic (*e.g*. RNA-seq data) or extrinsic (*e.g*. protein of close organisms) evidences. In order to provide an accurate gene prediction for each MAGs, we searched public databases for proteins from closely related organisms. For this, a MMseqs2^28^ database was created with proteins from UniRef90^38^ and METdb^39,40^. This database was aligned to the MAGs’ open-reading frames (min. 50 amino-acids) with mmseqs search (--start-sens 1 --sens-steps 3 -s 5 --translate 1 -e 0.00001 --min-length 50). For each MAG, the list of proteins with a match, hereafter referred as “protein hints”, were extracted with mmseqs convertalis followed by mmseqs createsubdb.

Two *ab initio* gene predictor were used. Augustus^41^ v3.5.0 was run with the protein hints aligned on each MAG using miniprot^42^ (introns up to 10 kb), while the best value for the --species parameter was estimated from a preliminary taxonomic annotation of the DNA-dependent RNA polymerase A, detected according to Delmont *et al*.^3^ methodology. Then, a custom gene model was generated per MAG with the scripts provided by Augustus, which was used to generate a final gene prediction. The genes were also predicted in a single pass by GeneMark-EP+^43^ v4.71, with protein hints aligned using ProtHint v2.6.0. Finally, both genes prediction were benchmarked with BUSCO^44^ v5.3.2 in “transcriptome*”* mode against the database eukaryota_odb10. The GeneMark-EP+ gene prediction strategy resulted in MAGs of higher average completeness (Augustus: 34,5%, GeneMark-EP+: 39,9%) with marginal increase in duplication rate (Fig. 3). Therefore, we retained the GeneMark-EP+ annotation as a reference.

### Single-cell sorting

Focussing on the small size fraction, subsamples of 50 mL were treated with 0.01% pluronic acid and diluted in TE buffer (10 mM Tris-HCl, 1 mM EDTA, pH 8, prefiltered to 0.2 μm), to achieve a flow rate of 1,500 events per second. These subsamples were stained with SYBR Green I (Thermo Fisher Scientific) at 1X final concentration for 10 min at room temperature in the dark.

Flow cytometric analysis and cell sorting were conducted using a BD FACSAria Fusion SORP cytometer installed in a Baker BioProtect IV BioSafety cabinet. The instrument was equipped with a 100 μm nozzle and a 1.5 neutral density filter, and operated using BD FACSDiva 9 software with sterile PBS as sheath fluid. Eukaryotic single cells were identified based on forward scatter (FSC), side scatter (SSC), and SYBR Green I fluorescence intensity, using a 488 nm (50 mW) laser with 502 nm longpass and 530/30 nm bandpass filters. Autotrophic and heterotrophic cells were distinguished by their chlorophyll content, detected using a 405 nm (50 mW) laser with 630 nm longpass and 660/20 nm bandpass filters.

Samples were analysed and sorted under sterile conditions at the lowest flow rate (maximum 1,500 events per second) in three successive stages: first in “continuous yield” mode, then in “continuous purity” mode, and finally in “single cell” mode. To promote cell settling at the bottom of the collection tubes, we sorted one cell together with 1000 Fluoresbrite YG Microspheres (0.50 μm; Polysciences, Warrington, PA, USA) into conical 96-well semi-skirted plates (Bio-Rad, Hercules, CA, USA) containing 5 μL of PBS. This resulted in an average amplification success rate of 30%.

### Single-cell genome amplification, screening, sequencing and assembly

Single-cell whole genome amplification was performed using the Single Cell GenomiPhi DNA Amplification kit (Cytiva, Marlborough, MA, USA) with minor modifications. Specifically, five freeze-thaw cycles (each lasting 4 min) were introduced prior to lysis with the kit’s lysis buffer. Additionally, the amplification reaction was extended to 16 h at 30 °C, instead of the standard 2 h.

The amplified genomic DNA was diluted 100-fold and used as a template for downstream PCR amplification. Nearly full-length 18S rDNA genes were amplified (95 °C for 5 min followed by 30 cycles 1 min-95 °C, 1 min-55 °C, 1 min-72 °C and a final extension 5 min-72 °C) using the eukaryotic primers Euk328f (5′-ACCTGGTTGATCCTGCCAG-3′)^45^ and Euk329r (5′-TGATCCTTCYGCAGGTTCAC-3′)^45^. PCR products were then cloned into pCRII-TOPO vectors (Invitrogen, Waltham, MA, USA) and transformed into *Escherichia coli* XL1-Blue competent cells^46^. Clones were randomly picked from different plates and checked for 18S rDNA inserts by PCR amplification using flanking vector primers (M13f and M13r). After purification (PCR purification kit; Qiagen), PCR product were sequenced (Sanger) by Eurofins and were compared to the NT database with the NCBI BLAST web application.

A total of 19 SAGs were selected for further sequencing, with a particular focus on potential parasites such as Perkinsea, Aphelida, and Chytrids. Fragmentation was started with 250 ng of DNA to reach a 100–1000 bp size range using the E220 Covaris (Covaris, Woburn, MA, USA) instrument. Illumina libraries were then prepared using the NEBNEXT End Repair, dA-Tailing and Quick Ligation modules (New England Biolabs) and the NEXTFLEX Unique Dual Index Barcodes (Perkin Elmer). Ligated products were purified by two consecutive 1X AMPure XP clean up (Beckman Coulter), then amplified by 10 PCR cycles using the KAPA HiFi HotStart Library Amplification Kit (Roche), amplification was then purified by 0.8X AMPure XP purification. Libraries were done in duplicates for four SAGs, resulting in 23 items. The libraries were sequenced with an Illumina NovaSeq 6000 instrument (2 × 150 bp).

The single-cell genomes were assembled with SPAdes^47^ v3.15.0 in single-cell mode (--sc). Contigs > 2.5 kb were kept and compared to the NT database by BLASTN^48^ *(*-evalue 1.10^-5^ -num_alignment 5 -outfmt “6 std staxid”) to identify and exclude contaminants (Human DNA, Bacteria, Archaea and Virus). Assembly quality was evaluated using QUAST^49^ and BUSCO^44^ v5.3.2 (-m genome and the eukaryota_odb10 dataset) and resulted in 14 assemblies corresponding to 11 SAGs that have been included in the dataset. Genes were predicted following the same strategy used for the MAGs with the difference that only GeneMark-EP+ was employed. SAG completion and redundancy were assessed with BUSCO^44^ v5.3.2 (protein mode) and eukaryota_odb10 dataset^31^.

### MAGs and SAGs tree based taxonomy and global distribution

The MAGs and SAGs taxonomy was further refined according to a phylogenomic approach. Following the methodology from Duncan *et al*.^4^, MAGs and SAGs proteins were first searched against the Phylosift^50^ v1.0.1 marker set (phylosift search --isolate --besthit), together with a selection of eukaryote reference genomes downloaded from the NCBI (datasets^51^ command-line tool, list of genomes in Data Record section for details). Fifty Phylosift markers which were present in at least 50% of the genomes were aligned (phylosift align --isolate --besthit). Trimmed alignments (trimAl^52^ v1.5 with --automated1) were concatenated for phylogenic inference with IQ-TREE^53^ v2.2.3 (-m LG + F + I + R10 -B 2000 --alrt 2000 --bnni).

MAGs and SAGs proteomes were also searched for the large and small DNA-dependent RNA polymerases subunits. Considering the DNA-dependent RNA polymerase genes reference dataset from Delmont *et al*.^3^, proteins were split into 6 files, one for each subunit^54^. The alignments of each subunit with MAFFT^55^ *v7.525* were cleaned with TrimAl^52^ v1.5 (--gt 0.5), and condensed in a HMM profile with hmmbuild^23^ v3.4. The 6 HMM profiles were searched against the MAGs, SAGs, the reference genomes used for the “Phylosift” analyses, plus the genomic reference database for marine species (METdb)^40^, and freshwater MAGs from Lake Mendota^5^ (hmmsearch v3.4*;* e-value threshold of 1e^-50^, retaining best-hit). Two sequences of the Malawimonada taxa were found directly in GenBank. Each marker was aligned separately with MAFFT^55^ v7.525, sites with more than 50% gaps were trimmed with goalign^56^ v0.3.7. Alignments were then concatenated and the tree was estimated by IQ-TREE^53^ v2.4.0 with the automatic model selection (-m MPF)^57^. Support was computed with 1,000 replicates for both the ultrafast bootstrap^58^ and the SH-like approximation likelihood ratio^59^. The taxonomy of MAGs and SAGs was inferred from their placement in the trees. Taxonomies of MAGs and SAGs recovered in both approaches were congruent, however the DNA-dependent RNA polymerase-based phylogeny (Fig. 3) was overall more resolutive.

The presence of our MAGs and SAGs was also investigated in about 3,000 public metagenomes that could contain eukaryotic data. The metagenome reads were filtered with fastp^60^ v0.23.4 authorising no N and an average quality greater than 30, prior their mapping with bwa mem^29^ v0.7.17, followed by samtools view^34^ (v1.16.1, parameters -F4 -q30 -bS) and samtools sort. Aligned reads were then filtered by msamtools filter^35^ v1.1.0 (-b -l 50 -p 95 -z 80). The coverage was calculated per contig with samtools coverage and merged per assembly afterwards. MAGs or SAGs with fewer than 1,500 reads mapped across all metagenomes were removed from the analysis, as well as metagenomes with less than 100 reads mapped. A MAG or SAG was considered detected in a metagenome when its breadth of coverage was at least 10%. To give some more insights on MAGs and SAGs shared distribution across different ecosystems, we performed metagenome clustering using read counts. The centered log ratio (CLR) transformation was applied to the mapped read count per metagenome on MAGs and SAGs (package clr^61^ v2.0-8 in R^62^ v4.4.1), with a preliminary replacement of zeros by the count zero multiplicative method (package zCompositions^63^ v1.5.0-4). Aitchison distance (*i.e*. Euclidian distance of CLR data) was computed and used for the metagenomes clustering with Ward.D2 method. The elbow method (fviz_nbclust()^64^ v1.0.7) was used to define optimal number of clusters, which was 3. These clusters have no apparent bias with either metagenome size or size fraction filtering (Figure S1).

## Data Records

All sequencing data were deposited in the ENA/SRA databases under the umbrella BioProject PRJEB61527^65^, which gather reads from the 18S rDNA V4 and V9 data (PRJEB61511^66^ and PRJEB94150^67^ respectively), the 18S rDNA V4 negative samples (PRJEB61512^68^), the metatranscriptomes (PRJEB61515^69^), the metagenomes (PRJEB94020^70^) and the SAGs reads (PRJEB94149^71^).

The other types of data generated for this article are stored in the Zenodo record 17812131^72^. It contains (i) the MAGs sequences and genes predictions (both with GeneMark and Augustus) in GFF3 format, the transcript and protein sequences as fasta files; (ii) the SAGs sequences; (iii) a table with MAGs and SAGs metadata (assembly statistics, BUSCO results, affiliation); (iv) the DNA-dependent RNA polymerase proteins HMM profiles from the 6 subunits used to build the dataset for the phylogeny, as well as their individuals and concatenated alignments; (v) the phylogeny built with Phylosift; (vi) a table with the MAGs and SAGs taxonomic annotations, (vii) the 4 metagenomic co-assemblies (contigs ≥ 2.5 kb) with the mapping of their reads and the MetaBAT2 bins, available as Anvi’o “contigs” and “profiles databases”, (viii) the unigenes annotations (taxonomic and functional) and the table of raw read count per sample, (ix) information about the public metagenomes that mapped on the MAGs and SAGs, in term of read count and genome detection (breadth of coverage), plus the metadata associated.

## Technical Validation

After extraction, the nucleic acids were quantified with the QuBit RNA HS and dsDNA HS assay kits (Thermo Fisher Scientific) on a Qubit model 3 fluorometer, and the RNA integrity was controlled with a TapeStation 2100 using the High Sensitivity RNA ScreenTape (Agilent, Santa Clara, CA, USA) and the absence of DNA was checked by PCR. RNA integrity numbers ranged from 7.3 to 9.4. For the DNA and RNA duplicates, 7 and 16 samples respectively failed to supply enough material and thus were pooled for sequencing.

The cell sorting required precautions. All equipment was sterilised by autoclaving or UV decontamination, the cytometer was cleaned and prepared for aseptic sorting according to the manufacturer’s instructions. The cytometer performance was verified and adjusted before each use with BD CS&T beads and drop delay was calibrated using BD FACS Accudrops Beads in autodelay mode. All signals were visualised on logarithmic scales, with the threshold set to the minimum value for the SYBR Green I parameter.

The sequencing platform (Genoscope) applied the following filters on the raw data, i) removing adapters and primers on the whole read and low quality nucleotides from both ends (Q-score lower than 20), ii) removing sequences between the second unknown nucleotide (N) and the end of the read, iii) discarding reads shorter than 30 nucleotides after trimming and iv) removing read pairs that come from the low-concentration spike-in library of Illumina PhiX Control. The small contigs (≤2.5 kb) were removed from the assemblies as advised by MetaBAT2^36^. Reads were mapped back on the assembly and alignments were filtered to retain those greater than 95% identity on at least 75 nt and the highest scoring hit was kept when multi-mapping occurred. MAGs and SAGs completion and redundancy scores were estimated by BUSCO and the dataset “eukaryota_odb10”. The metatranscriptomic libraries were subject to rDNA (5S, 5.8S, 16S, 18S, 23S and 28S) contamination evaluation. The unigenes are the representative sequences from transcripts clustered by CD-HIT-EST >95% identity and >90% of the length of the smallest sequence. For the 18S rDNA V4 metabarcoding, a negative control was sequenced and the raw reads were deposited under the BioProject PRJEB61512^68^.

## Supplementary information

Supplementary Information

## Data Availability

All sequencing data were deposited in the ENA/SRA databases under the BioProjects PRJEB61527^65^ (Umbrella), PRJEB61511^66^, PRJEB61512^68^, PRJEB61515^69^, PRJEB94020^70^, PRJEB94149^71^ and PRJEB94150^67^. The processed data are available in the Zenodo record 17812131^72^.

## Code availability

Scripts related to the metabarcoding and metatrancriptomics data processing are available in the GitHub repository https://github.com/meb-team/MICROSTORE_MetaT-MetaB_Monjot_2023. Examples of codes and scripts used to process the metagenomic co-assemblies, the gene prediction, and for the mapping of public metagenomes, are available in the repository https://github.com/meb-team/datapaper_eukaryotes_pavin_2025.

## References

[1] Wu, D., Seshadri, R., Kyrpides, N. C. & Ivanova, N. N. A metagenomic perspective on the microbial prokaryotic genome census. *Sci. Adv*. **11**, 10.1126/sciadv.adq2166 (2025). 10.1126/sciadv.adq2166 PMC11740963 39823337

[2] Wilken, S., Choi, C. J. & Worden, A. Z. Contrasting Mixotrophic Lifestyles Reveal Different Ecological Niches in Two Closely Related Marine Protists. *J. Phycol.* **56**, 52–67, 10.1111/jpy.12920 (2020). 31529498 10.1111/jpy.12920 PMC7065223

[3] Delmont, T. O. *et al*. Functional repertoire convergence of distantly related eukaryotic plankton lineages abundant in the sunlit ocean. *Cell Genomics* **2**, 100123, 10.1016/j.xgen.2022.100123 (2022). 36778897 10.1016/j.xgen.2022.100123 PMC9903769

[4] Duncan, A. *et al*. Metagenome-assembled genomes of phytoplankton microbiomes from the Arctic and Atlantic Oceans. *Microbiome* **10**, 67, 10.1186/s40168-022-01254-7 (2022). 35484634 10.1186/s40168-022-01254-7 PMC9047304

[5] Krinos, A. I. *et al*. Time-series metagenomics reveals changing protistan ecology of a temperate dimictic lake. *Microbiome* **12**, 133, 10.1186/s40168-024-01831-y (2024). 39030632 10.1186/s40168-024-01831-y PMC11265017

[6] Boukheloua, R. *et al*. Global freshwater distribution of Telonemia protists. *ISME J.* **18**, wrae177, 10.1093/ismejo/wrae177 (2024). 39303138 10.1093/ismejo/wrae177 PMC11512789

[7] *Lake Pavin*, 10.1007/978-3-319-39961-4 (Springer International Publishing, Cham, 2016).

[8] Eren, A. M. *et al*. Anvi’o: an advanced analysis and visualization platform for ‘omics data. *PeerJ* **3**, e1319, 10.7717/peerj.1319 (2015). 26500826 10.7717/peerj.1319 PMC4614810

[9] Carradec, Q. *et al*. A global ocean atlas of eukaryotic genes. *Nat. Commun.* **9**, 373, 10.1038/s41467-017-02342-1 (2018). 29371626 10.1038/s41467-017-02342-1 PMC5785536

[10] Monjot, A. *et al*. Functional diversity of microbial eukaryotes in a meromictic lake: Coupling between metatranscriptomic and a trait‐based approach. *Environ. Microbiol.* **25**, 3406–3422, 10.1111/1462-2920.16531 (2023). 37916456 10.1111/1462-2920.16531

[11] Monjot, A., Rousseau, J., Bittner, L. & Lepère, C. Metatranscriptomes-based sequence similarity networks uncover genetic signatures within parasitic freshwater microbial eukaryotes. *Microbiome* **13**, 43, 10.1186/s40168-024-02027-0 (2025). 39915863 10.1186/s40168-024-02027-0 PMC11800578

[12] Schoenle, A. *et al*. Protist genomics: key to understanding eukaryotic evolution. *Trends Genet*. 10.1016/j.tig.2025.05.004 (2025). 10.1016/j.tig.2025.05.004 40517085

[13] Parada, A. E., Needham, D. M. & Fuhrman, J. A. Every base matters: assessing small subunit rRNA primers for marine microbiomes with mock communities, time series and global field samples. *Environ. Microbiol.* **18**, 1403–1414, 10.1111/1462-2920.13023 (2016). 26271760 10.1111/1462-2920.13023

[14] Mangot, J.-F. *et al*. Short-term dynamics of diversity patterns: evidence of continual reassembly within lacustrine small eukaryotes. *Environ. Microbiol.* **15**, 1745–1758, 10.1111/1462-2920.12065 (2013). 23297806 10.1111/1462-2920.12065

[15] Chauvet, M., Debroas, D., Moné, A., Dubuffet, A. & Lepère, C. Temporal variations of Microsporidia diversity and discovery of new host–parasite interactions in a lake ecosystem. *Environ. Microbiol.* **24**, 1672–1686, 10.1111/1462-2920.15950 (2022). 35246918 10.1111/1462-2920.15950

[16] Amaral-Zettler, L. A., McCliment, E. A., Ducklow, H. W. & Huse, S. M. A Method for Studying Protistan Diversity Using Massively Parallel Sequencing of V9 Hypervariable Regions of Small-Subunit Ribosomal RNA Genes. *PLOS ONE* **4**, e6372, 10.1371/journal.pone.0006372 (2009). 19633714 10.1371/journal.pone.0006372 PMC2711349

[17] López-García, P., Philippe, H., Gail, F. & Moreira, D. Autochthonous eukaryotic diversity in hydrothermal sediment and experimental microcolonizers at the Mid-Atlantic Ridge. *Proc. Natl. Acad. Sci.* **100**, 697–702, 10.1073/pnas.0235779100 (2003). 12522264 10.1073/pnas.0235779100 PMC141059

[18] Zerbino, D. R. & Birney, E. Velvet: algorithms for de novo short read assembly using de Bruijn graphs. *Genome Res.* **18**, 821–829, 10.1101/gr.074492.107 (2008). 18349386 10.1101/gr.074492.107 PMC2336801

[19] Schulz, M. H., Zerbino, D. R., Vingron, M. & Birney, E. Oases: robust de novo RNA-seq assembly across the dynamic range of expression levels. *Bioinformatics* **28**, 1086–1092, 10.1093/bioinformatics/bts094 (2012). 22368243 10.1093/bioinformatics/bts094 PMC3324515

[20] Fu, L., Niu, B., Zhu, Z., Wu, S. & Li, W. CD-HIT: accelerated for clustering the next-generation sequencing data. *Bioinformatics*** 28**, 3150–3152, 10.1093/bioinformatics/bts565 (2012). 10.1093/bioinformatics/bts565 PMC3516142 23060610

[21] Haas, B. J. TransDecoder, https://github.com/TransDecoder/TransDecoder (2018).

[22] Eberhardt, R. Y. *et al*. AntiFam: a tool to help identify spurious ORFs in protein annotation. *Database* **2012**, bas003, 10.1093/database/bas003 (2012). 22434837 10.1093/database/bas003 PMC3308159

[23] Eddy, S. R. Accelerated Profile HMM Searches. *PLOS Comput. Biol.* **7**, e1002195, 10.1371/journal.pcbi.1002195 (2011). 22039361 10.1371/journal.pcbi.1002195 PMC3197634

[24] Aramaki, T. *et al*. KofamKOALA: KEGG Ortholog assignment based on profile HMM and adaptive score threshold. *Bioinformatics* **36**, 2251–2252, 10.1093/bioinformatics/btz859 (2020). 31742321 10.1093/bioinformatics/btz859 PMC7141845

[25] Mistry, J. *et al*. Pfam: The protein families database in 2021. *Nucleic Acids Res.* **49**, D412–D419, 10.1093/nar/gkaa913 (2021). 33125078 10.1093/nar/gkaa913 PMC7779014

[26] Gene Ontology Consortium. Pfam2GO. 10.5281/zenodo.6399963 (2022).

[27] Levy Karin, E., Mirdita, M. & Söding, J. MetaEuk—sensitive, high-throughput gene discovery, and annotation for large-scale eukaryotic metagenomics. *Microbiome* **8**, 48, 10.1186/s40168-020-00808-x (2020). 32245390 10.1186/s40168-020-00808-x PMC7126354

[28] Steinegger, M. & Söding, J. MMseqs2 enables sensitive protein sequence searching for the analysis of massive data sets. *Nat. Biotechnol.* **35**, 1026–1028, 10.1038/nbt.3988 (2017). 29035372 10.1038/nbt.3988

[29] Li, H. Aligning sequence reads, clone sequences and assembly contigs with BWA-MEM. Preprint at 10.48550/arXiv.1303.3997 (2013).

[30] Benoit, G. *et al*. SimkaMin: fast and resource frugal de novo comparative metagenomics. *Bioinformatics* **36**, 1275–1276, 10.1093/bioinformatics/btz685 (2020). 31504187 10.1093/bioinformatics/btz685

[31] Li, D., Liu, C.-M., Luo, R., Sadakane, K. & Lam, T.-W. MEGAHIT: an ultra-fast single-node solution for large and complex metagenomics assembly via succinct de Bruijn graph. *Bioinformatics* **31**, 1674–1676, 10.1093/bioinformatics/btv033 (2015). 25609793 10.1093/bioinformatics/btv033

[32] Hyatt, D. *et al*. Prodigal: prokaryotic gene recognition and translation initiation site identification. *BMC Bioinformatics* **11**, 119, 10.1186/1471-2105-11-119 (2010). 20211023 10.1186/1471-2105-11-119 PMC2848648

[33] Li, H. & Durbin, R. Fast and accurate short read alignment with Burrows–Wheeler transform. *Bioinformatics* **25**, 1754–1760, 10.1093/bioinformatics/btp324 (2009). 19451168 10.1093/bioinformatics/btp324 PMC2705234

[34] Danecek, P. *et al*. Twelve years of SAMtools and BCFtools. *GigaScience* **10**, giab008, 10.1093/gigascience/giab008 (2021). 33590861 10.1093/gigascience/giab008 PMC7931819

[35] Arumugam, M. msamtools, https://github.com/arumugamlab/msamtools (2025).

[36] Kang, D. D. *et al*. MetaBAT 2: an adaptive binning algorithm for robust and efficient genome reconstruction from metagenome assemblies. *PeerJ* **7**, e7359, 10.7717/peerj.7359 (2019). 31388474 10.7717/peerj.7359 PMC6662567

[37] Delmont, T. O. Assessing the completion of eukaryotic bins with anvi’o. *Meren Lab* https://merenlab.org/2018/05/05/eukaryotic-single-copy-core-genes/ (2018).

[38] Suzek, B. E., Huang, H., McGarvey, P., Mazumder, R. & Wu, C. H. UniRef: comprehensive and non-redundant UniProt reference clusters. *Bioinformatics* **23**, 1282–1288, 10.1093/bioinformatics/btm098 (2007). 17379688 10.1093/bioinformatics/btm098

[39] Keeling, P. J. *et al*. The Marine Microbial Eukaryote Transcriptome Sequencing Project (MMETSP): Illuminating the Functional Diversity of Eukaryotic Life in the Oceans through Transcriptome Sequencing. *PLOS Biol.* **12**, e1001889, 10.1371/journal.pbio.1001889 (2014). 24959919 10.1371/journal.pbio.1001889 PMC4068987

[40] Niang, G. *et al*. METdb: A Genomic Reference Database for Marine Species. *F1000Research* **9**, 10.7490/f1000research.1118000.1 (2020).

[41] Stanke, M. *et al*. AUGUSTUS: ab initio prediction of alternative transcripts. *Nucleic Acids Res.* **34**, W435–W439, 10.1093/nar/gkl200 (2006). 16845043 10.1093/nar/gkl200 PMC1538822

[42] Li, H. Protein-to-genome alignment with miniprot. *Bioinformatics* **39**, btad014, 10.1093/bioinformatics/btad014 (2023). 36648328 10.1093/bioinformatics/btad014 PMC9869432

[43] Brůna, T., Lomsadze, A. & Borodovsky, M. GeneMark-EP+: eukaryotic gene prediction with self-training in the space of genes and proteins. *NAR Genomics Bioinforma.* **2**, lqaa026, 10.1093/nargab/lqaa026 (2020). 10.1093/nargab/lqaa026 PMC7222226 32440658

[44] Manni, M., Berkeley, M. R., Seppey, M., Simão, F. A. & Zdobnov, E. M. BUSCO Update: Novel and Streamlined Workflows along with Broader and Deeper Phylogenetic Coverage for Scoring of Eukaryotic, Prokaryotic, and Viral Genomes. *Mol. Biol. Evol.* **38**, 4647–4654, 10.1093/molbev/msab199 (2021). 34320186 10.1093/molbev/msab199 PMC8476166

[45] Moon-van der Staay, S. Y. *et al*. Abundance and Diversity of Prymnesiophytes in the Picoplankton Community from the Equatorial Pacific Ocean Inferred from 18S rDNA Sequences. *Limnol. Oceanogr.* **45**, 98–109 (2000).

[46] Inoue, H., Nojima, H. & Okayama, H. High efficiency transformation of *Escherichia coli* with plasmids. *Gene* **96**, 23–28, 10.1016/0378-1119(90)90336-P (1990). 2265755 10.1016/0378-1119(90)90336-p

[47] Prjibelski, A., Antipov, D., Meleshko, D., Lapidus, A. & Korobeynikov, A. Using SPAdes De Novo Assembler. *Curr. Protoc. Bioinforma.* **70**, e102, 10.1002/cpbi.102 (2020). 10.1002/cpbi.102 32559359

[48] Camacho, C. *et al*. BLAST+: architecture and applications. *BMC Bioinformatics* **10**, 421, 10.1186/1471-2105-10-421 (2009). 20003500 10.1186/1471-2105-10-421 PMC2803857

[49] Mikheenko, A., Prjibelski, A., Saveliev, V., Antipov, D. & Gurevich, A. Versatile genome assembly evaluation with QUAST-LG. *Bioinformatics* **34**, i142–i150, 10.1093/bioinformatics/bty266 (2018). 10.1093/bioinformatics/bty266 PMC6022658 29949969

[50] Darling, A. E. *et al*. PhyloSift: phylogenetic analysis of genomes and metagenomes. *PeerJ* **2**, e243, 10.7717/peerj.243 (2014). 24482762 10.7717/peerj.243 PMC3897386

[51] O’Leary, N. A. *et al*. Exploring and retrieving sequence and metadata for species across the tree of life with NCBI Datasets. *Sci. Data* **11**, 732, 10.1038/s41597-024-03571-y (2024). 38969627 10.1038/s41597-024-03571-y PMC11226681

[52] Capella-Gutiérrez, S., Silla-Martínez, J. M. & Gabaldón, T. trimAl: a tool for automated alignment trimming in large-scale phylogenetic analyses. *Bioinformatics* **25**, 1972–1973, 10.1093/bioinformatics/btp348 (2009). 19505945 10.1093/bioinformatics/btp348 PMC2712344

[53] Minh, B. Q. *et al*. IQ-TREE 2: New Models and Efficient Methods for Phylogenetic Inference in the Genomic Era. *Mol. Biol. Evol.* **37**, 1530–1534, 10.1093/molbev/msaa015 (2020). 32011700 10.1093/molbev/msaa015 PMC7182206

[54] Werner, F. & Grohmann, D. Evolution of multisubunit RNA polymerases in the three domains of life. *Nat. Rev. Microbiol.* **9**, 85–98, 10.1038/nrmicro2507 (2011). 21233849 10.1038/nrmicro2507

[55] Katoh, K. & Standley, D. M. MAFFT Multiple Sequence Alignment Software Version 7: Improvements in Performance and Usability. *Mol. Biol. Evol.* **30**, 772–780, 10.1093/molbev/mst010 (2013). 23329690 10.1093/molbev/mst010 PMC3603318

[56] Lemoine, F. & Gascuel, O. Gotree/Goalign: toolkit and Go API to facilitate the development of phylogenetic workflows. *NAR Genomics Bioinforma.* **3**, lqab075, 10.1093/nargab/lqab075 (2021). 10.1093/nargab/lqab075 PMC8356961 34396097

[57] Kalyaanamoorthy, S., Minh, B. Q., Wong, T. K. F., von Haeseler, A. & Jermiin, L. S. ModelFinder: fast model selection for accurate phylogenetic estimates. *Nat. Methods* **14**, 587–589, 10.1038/nmeth.4285 (2017). 28481363 10.1038/nmeth.4285 PMC5453245

[58] Minh, B. Q., Nguyen, M. A. T. & von Haeseler, A. Ultrafast Approximation for Phylogenetic Bootstrap. *Mol. Biol. Evol.* **30**, 1188–1195, 10.1093/molbev/mst024 (2013). 23418397 10.1093/molbev/mst024 PMC3670741

[59] Guindon, S. *et al*. New Algorithms and Methods to Estimate Maximum-Likelihood Phylogenies: Assessing the Performance of PhyML 3.0. *Syst. Biol.* **59**, 307–321, 10.1093/sysbio/syq010 (2010). 20525638 10.1093/sysbio/syq010

[60] Chen, S. Ultrafast one-pass FASTQ data preprocessing, quality control, and deduplication using fastp. *iMeta* **2**, e107, 10.1002/imt2.107 (2023). 38868435 10.1002/imt2.107 PMC10989850

[61] Boogaart, K. G., van den, Tolosana-Delgado, R. & Bren, M. *Compositions: Compositional Data Analysis*10.32614/CRAN.package.compositions (2024).

[62] R Core Team. R: A Language and Environment for Statistical Computing, https://www.R-project.org/ (R Foundation for Statistical Computing, Vienna, Austria, 2024).

[63] Palarea-Albaladejo, J. & Martín-Fernández, J. A. zCompositions – R package for multivariate imputation of left-censored data under a compositional approach. *Chemom. Intell. Lab. Syst.* **143**, 85–96, 10.1016/j.chemolab.2015.02.019 (2015).

[64] Kassambara, A. & Mundt, F. Factoextra: Extract and Visualize the Results of Multivariate Data Analyses, 10.32614/CRAN.package.factoextra (2020).

[65] *ENA European Nucleotide Archive* https://identifiers.org/ena.embl:PRJEB61527 (2023). 10.1093/nar/gkad1067 PMC10767888 37956313

[66] *ENA European Nucleotide Archive* https://identifiers.org/ena.embl:PRJEB61511 (2023). 10.1093/nar/gkad1067 PMC10767888 37956313

[67] *ENA European Nucleotide Archive* https://identifiers.org/ena.embl:PRJEB94150 (2025). 10.1093/nar/gkaf1295 PMC12807680 41335099

[68] *ENA European Nucleotide Archive* https://identifiers.org/ena.embl:PRJEB61512 (2023). 10.1093/nar/gkad1067 PMC10767888 37956313

[69] *ENA European Nucleotide Archive* https://identifiers.org/ena.embl:PRJEB61515 (2023). 10.1093/nar/gkad1067 PMC10767888 37956313

[70] *ENA European Nucleotide Archive* https://identifiers.org/ena.embl:PRJEB94020 (2025). 10.1093/nar/gkaf1295 PMC12807680 41335099

[71] *ENA European Nucleotide Archive* https://identifiers.org/ena.embl:PRJEB94149 (2025). 10.1093/nar/gkaf1295 PMC12807680 41335099

[72] Courtine, D. *et al*. MICROSTORE:’omic approaches to decipher MICrobial eukaRyOteS funcTiOn in fReshwater lake Ecosystems. *Zenodo*10.5281/zenodo.17812131 (2025).

