# Supplementary material for: A multi-Omic resource for exploring microbial eukaryotes in the meromictic freshwater Lake Pavin
Source: Sci Data. 2026 Jan 14;13:252. doi: 10.1038/s41597-026-06573-0 (PMC12913720; doi:10.1038/s41597-026-06573-0)
Supplement: Supplementary file 1 — Supplementary Information [file 41597_2026_6573_MOESM1_ESM.pdf]

UMAP based on CLR-transformed raw read cound of metagenomes mapped on MAGs and SAGs

Parameters : 25 neighbors, minimal distance 0.25

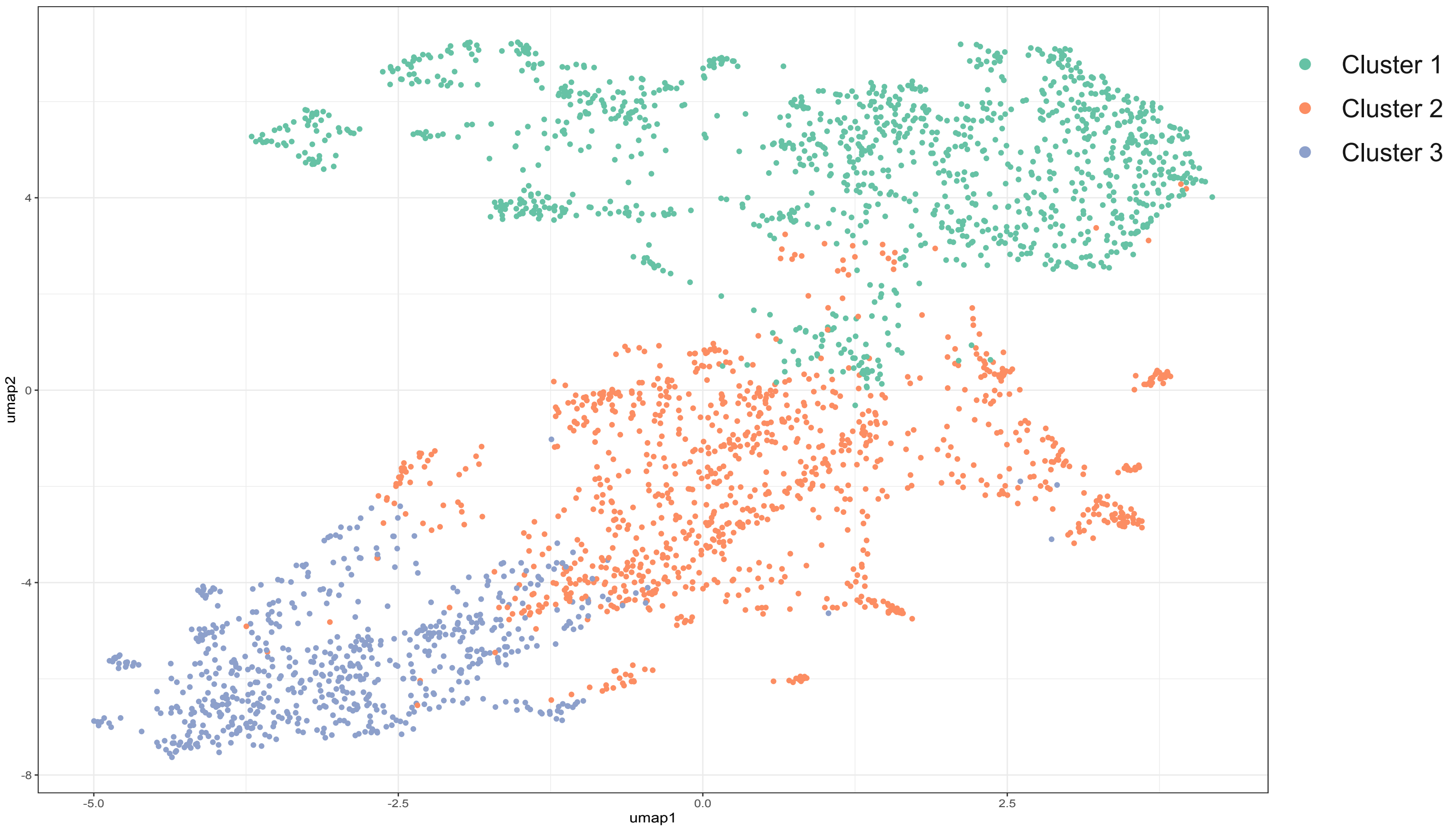

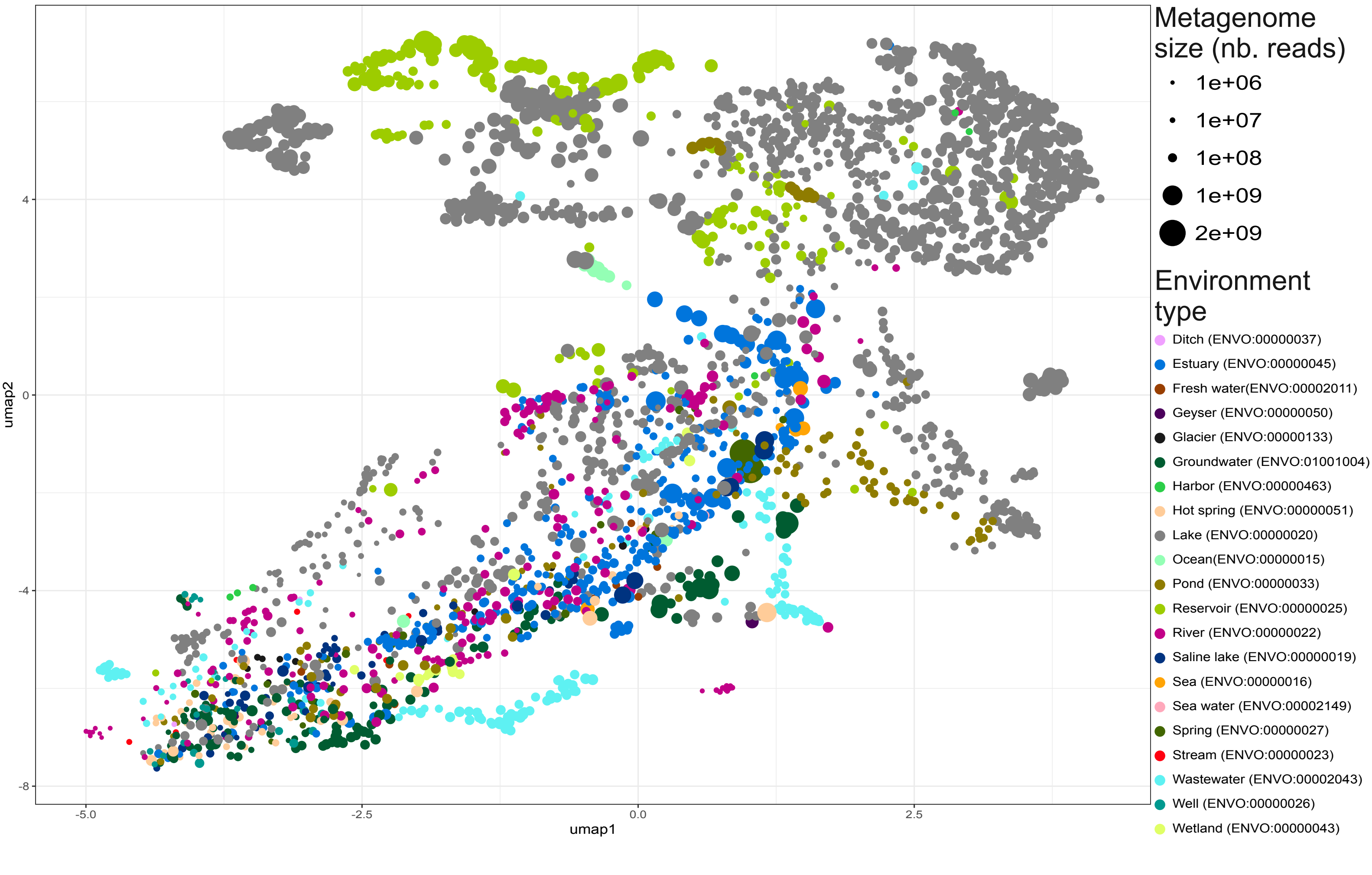

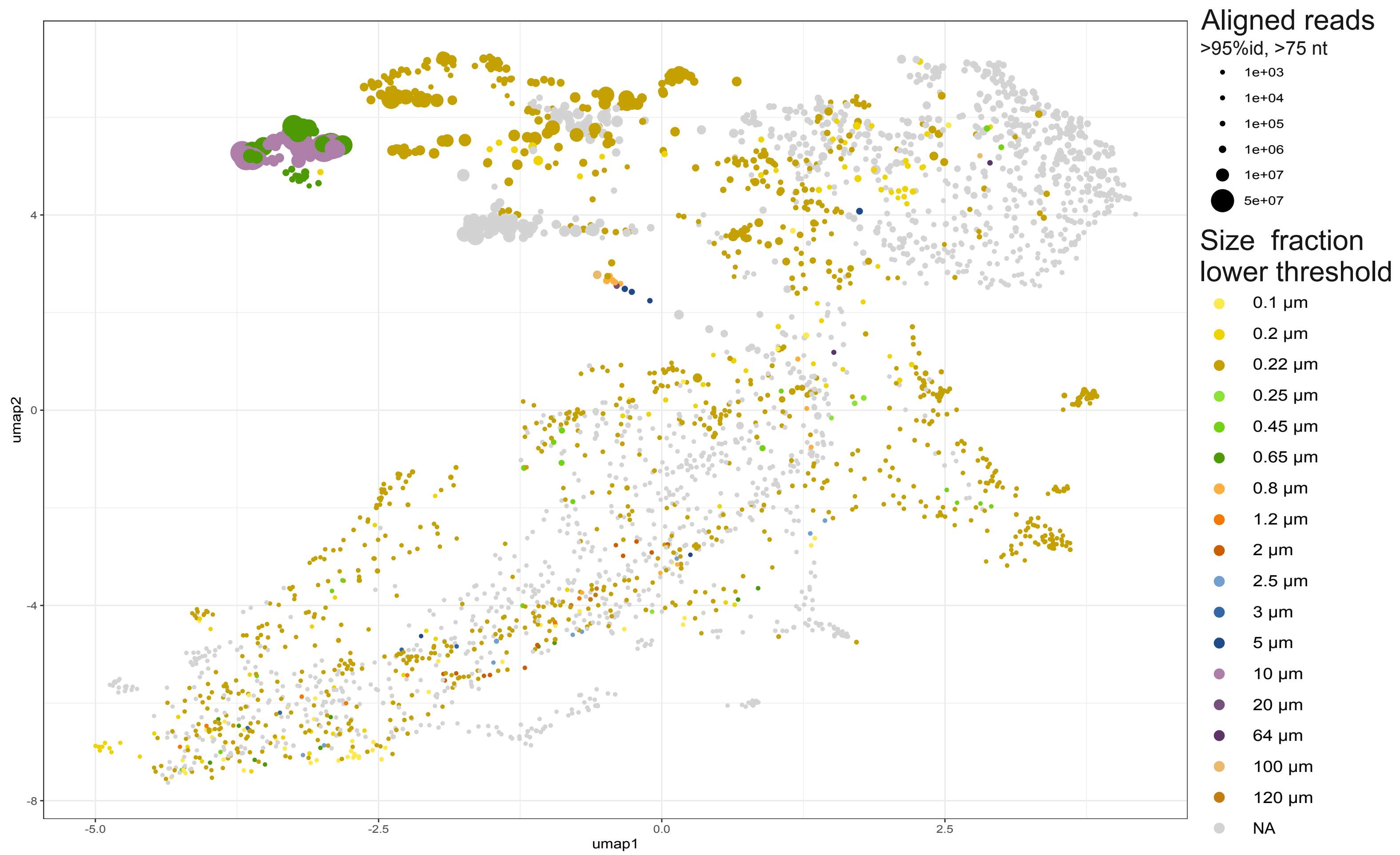
Aligned reads
>95%id, >75 nt
1e+03
1e+04
1e+05
1e+06
1e+07
5e+07
Size fraction
lower threshold
0.1 µm
0.2 µm
0.22 µm
0.25 µm
0.45 µm
0.65 µm
0.8 µm
1.2 µm
2 µm
2.5 µm
3 µm
5 µm
10 µm
20 µm
64 µm
100 µm
120 µm
NA
umap1
umap2
-5.0
-2.5
0.0
2.5
4
0
-4
-8

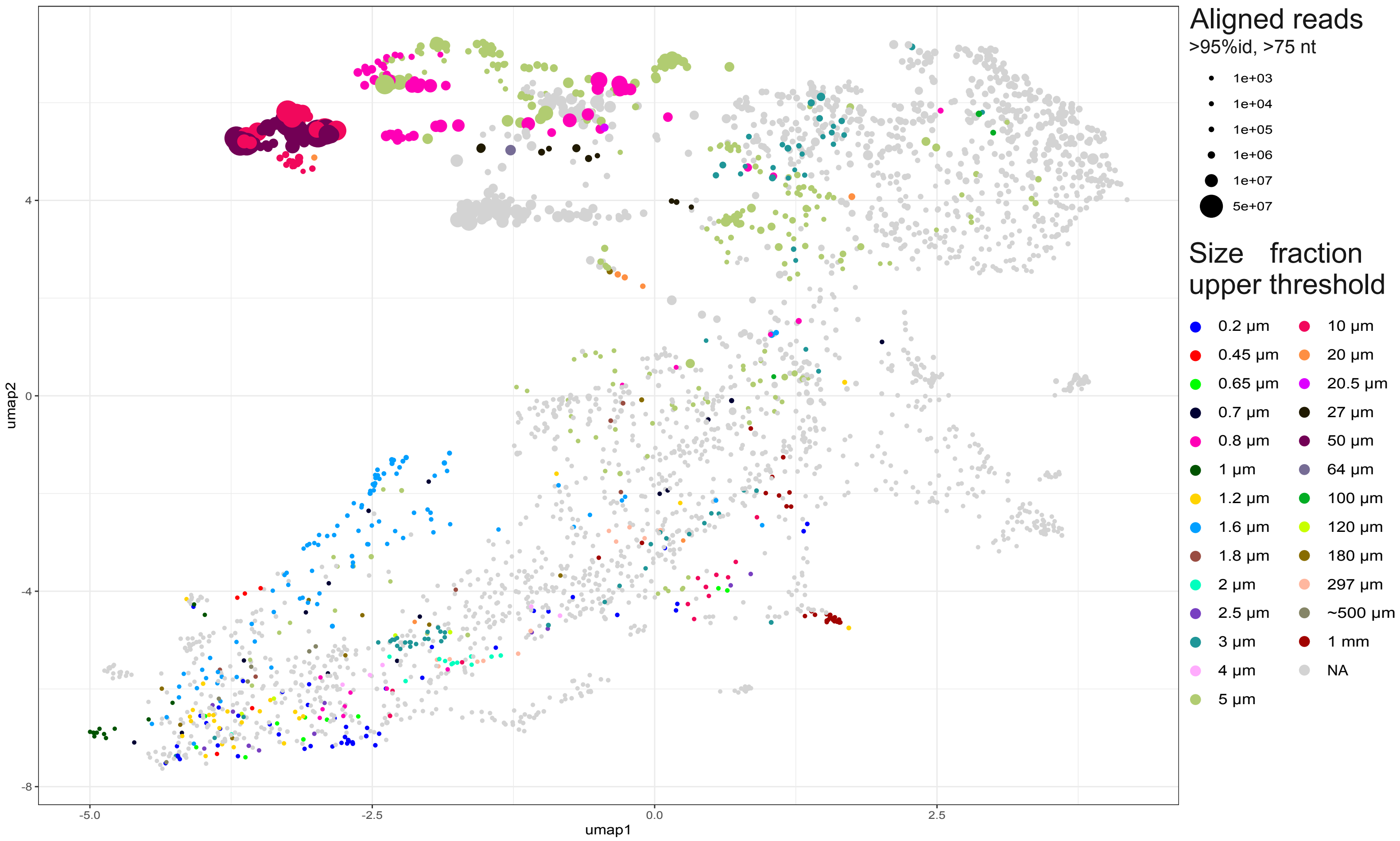
Aligned reads
>95%id, >75 nt
1e+03
1e+04
1e+05
1e+06
1e+07
5e+07
Size fraction
upper threshold
0.2 µm
0.45 µm
0.65 µm
0.7 µm
0.8 µm
1 µm
1.2 µm
1.6 µm
1.8 µm
2 µm
2.5 µm
3 µm
4 µm
5 µm
10 µm
20 µm
20.5 µm
27 µm
50 µm
64 µm
100 µm
120 µm
180 µm
297 µm
~500 µm
1 mm
NA
umap1
umap2
-5.0
-2.5
0.0
2.5
4
0
-4
-8

Supplementary Figure 1: UMAP dimension reduction of CLR-transformed raw read count from metagenomes mapped on MAGs and SAGs.

All panels represent the same UMAP, with different associated metadata. The UMAP was constructed with 25 neighbors and a minimal distance of 0.25, based on the CLR-transformed read count, with the UMAP *R* package[1]. a, clusters of metagenomes based on the Aitchison distance (Euclidean distance on CLR-transformed data). b, the size (number of reads) and the environment type of each metagenomic dataset. c, the lower threshold of the size fraction (*i.e.* the filter size on which the sample was collected), and the number of read that aligned of the MAGs and SAGs. d, the upper threshold of the size fraction.

1. Konopka, T. Umap: Uniform Manifold Approximation and Projection. (2023).
